# Accuracy of autorefraction in an adult Indian population

**DOI:** 10.1371/journal.pone.0251583

**Published:** 2021-05-19

**Authors:** Rajesh S. Kumar, Caitlin A. Moe, Deepak Kumar, Mahalakshmi V. Rackenchath, Sathi Devi A. V., Sriharsha Nagaraj, Dionna M. Wittberg, Robert L. Stamper, Jeremy D. Keenan

**Affiliations:** 1 Narayana Nethralaya Eye Hospital, Bangalore, India; 2 Cleveland Clinic Abu Dhabi, Abu Dhabi, UAE; 3 Francis I Proctor Foundation, University of California, San Francisco, San Francisco, CA, United States of America; 4 Department of Ophthalmology, University of California, San Francisco, San Francisco, CA, United States of America; Faculty of Medicine, Cairo University, EGYPT

## Abstract

**Purpose:**

Autorefractors allow non-specialists to quickly assess refractive error, and thus could be a useful component of large-scale vision screening programs. In order to better characterize the role of autorefraction for public health outreach programs in resource-limited settings, the diagnostic accuracy of two autorefractors was assessed relative to subjective refraction in an adult Indian population.

**Methods:**

An optometrist refracted a series of patients aged ≥50 years at an eye clinic in Bangalore, India using the Nidek ARK-900 autorefractor first, followed by the 3nethra Royal autorefractor, and then subjective refraction. The diagnostic accuracy of each autorefractor for myopia, hyperopia, and astigmatism was assessed using subjective refraction as the reference standard, and measures of agreement between refractions were calculated.

**Results:**

A total of 197 eyes in 104 individuals (mean age 63 ± 8 years, 52% female) were evaluated. Both autorefractors produced spherical equivalent estimates that were on average more hyperopic than subjective refraction, with a measurement bias of +0.16 D (95%CI +0.09 to +0.23D) for Nidek and +0.42 D (95%CI +0.28 to +0.54D) for 3nethra. When comparing pairs of measurements from autorefraction and subjective refraction, the limits of agreement were approximately ±1D for the Nidek autorefractor and ±1.75D for the 3Nethra autorefractor. The sensitivity and specificity of detecting ≥1 diopter of myopia were 94.6% (95%CI 86.8–100%) and 92.5% (95%CI 88.9–97.5%) for the Nidek, and 89.2% (95%CI 66.7–97.4) and 77.5% (95%CI 71.2–99.4%) for the 3Nethra. The accuracy of each autorefractor increased at greater levels of refractive error.

**Conclusions:**

The sensitivity and specificity of the Nidek autorefractor for diagnosing refractive error among adults ≥50 years in an urban Indian clinic was sufficient for screening for visually significant refractive errors, although the relatively wide limits of agreement suggest that subjective refinement of the eyeglasses prescription would still be necessary.

## Introduction

Uncorrected refractive error is the leading cause of visual impairment worldwide, with the greatest burden of disease in low and middle-income countries [1]. Many eye hospitals in low-resource settings conduct community-based outreach activities largely focused on detection of cataract and refractive error [1]. Such outreach programs often deploy trained refractionists to the community, where they perform subjective refraction with trial lenses. This strategy requires a sufficient supply of refractionists, making it unrealistic in many low-resource settings. An alternative strategy could employ autorefraction, which requires far less training, and thus would greatly increase the availability of refractive error screening. Such a program could greatly increase the availability of spectacles in the developing world, but would be dependent on accurate autorefraction. However, the applicability of autorefraction for screening activities is unclear since its diagnostic accuracy has typically been studied in children and younger adults [2–13], with considerably fewer studies including older adults [14–18]. An autorefractor would ideally give a result similar to subjective refraction, but subjective refraction is inherently a psychophysical procedure that relies on the cognitive ability and participation of the subject being tested. Thus, studies of older adults are important to assess the accuracy of autorefraction for screening older adults for refractive error. In this study we performed subjective refraction and autorefraction on a population of Indian adults aged 50 years and over to assess the accuracy of autorefraction relative to subjective refraction.

## Materials and methods

### Ethics

Ethical approval was obtained from the institutional review boards at the University of California, San Francisco (#13–10776) and Narayana Nethralaya Eye Hospital, Bangalore, India (#C/2013/06/03). Written informed consent was obtained from all participants and the study was conducted within the Tenets of the Declaration of Helsinki.

### Study design

A prospective consecutive series of patients aged 50 years or older visiting the comprehensive eye clinic at Narayana Nethralaya Eye Hospital (Bangalore, India) in September 2015 was offered enrollment in this study. Patients were eligible for the study if they were able to undergo all three refraction tests; eyes with best corrected visual acuity of 20/200 or worse after subjective refraction were excluded since it was deemed that subjective refraction would be difficult at such poor visual acuity. Refractive error was assessed with the Nidek ARK-900 (Nidek Co., LTD., Tokyo, Japan), then the 3nethra Royal (Forus Health Inc.; Bangalore, India), and finally by retinoscopy followed by subjective refraction. All refractions were performed without cycloplegia by the same experienced optometrist. The 3nethra device was chosen since it included both an autorefractor and fundus camera and was thus well-suited for mobile screening activities. The Nidek machine was the autorefractor routinely used in the clinic.

### Refraction calculations

Refraction measurements were transformed using Fourier vector decomposition to spherical equivalent and Jackson cross cylinder scalar values using the following formulas, where S = sphere, C = cylinder, and θ = axis in plus-cylinder clinical notation, SE indicates the spherical equivalent, J_0_ represents the power of the Jackson cross cylinder at 0°, and J_45_ represents the power of the Jackson cross cylinder at 45° [19, 20]:

$$SE=S+\frac{C}{2}$$

$$J_{0}=-\frac{C}{2}cos2\theta$$

$$J_{45}=-\frac{C}{2}sin2\theta$$

### Statistical methods

Agreement between each auto-refractor and subjective refraction was assessed by calculating the Bland-Altman 95% limits of agreement and intraclass correlation coefficient (ICC; modeled with absolute agreement and one-way random effects). The diagnostic accuracy of each autorefractor for myopia, hyperopia, and astigmatism was assessed assuming subjective refraction as the reference standard. Subjective refraction was chosen as the reference standard because it is currently the test generally used to dispense eyeglasses. In exploratory analyses, receiver operating characteristic (ROC) curves were constructed and areas under the curve (AUC) determined over a range of definitions of refractive error. Optimal diagnostic thresholds from ROC curves were calculated with the Youden method, which maximizes the sum of sensitivity and specificity. The mean refraction measurements were compared between each autorefractor and subjective refraction using a paired t-test. Analyses are reported with bootstrapped 95% confidence intervals with resampling at the participant level to account for nonindependence of eyes from the same person. None of the test results were indeterminate or missing. A sample size of 100 eyes would allow a confidence interval of ±11% for sensitivity, assuming one-third of participants had refractive error and a sensitivity of 90%; given the correlation between two eyes from the same person we targeted a sample size of approximately double this amount. All analyses were performed with Stata 14 (StataCorp LP; College Station, TX).

## Results

Study participants were enrolled in September 2015. A total of 198 eyes from 104 participants had all 3 refraction tests successfully performed. No adverse events occurred during testing. One eye had a best corrected visual acuity of 20/200 or worse and was excluded. Approximately half of the 104 study participants were female (N = 54; 52%) and the average age of participants was 63 (standard deviation 8 years, range 50 to 90 years). Table 1 shows eye-level characteristics. After subjective refraction, 37 of 197 eyes (19%) had 1 or more diopters of myopic spherical equivalent, 28 (14%) had 1 or more diopters of hyperopic spherical equivalent, and 77 (39%) had 1 or more diopters of astigmatism. BCVA with the subjective refraction was 20/40 or better in 176 eyes (89%), the remaining 21 eyes (9%) had a BCVA between 20/50 and 20/80. The mean scalar values of the decomposed vectors (i.e, spherical equivalent, J0, and J45) from each refraction method are shown in Table 1.

**Table 1:** Refractive characteristics of 197 study eyes.

Characteristic	N (%)	Mean diopters (95%CI)
Subjective Refraction		
Spherical equivalent category		
≤ -2.00 D	16 (8%)	
-1.875 to -1.00 D	21 (11%)	
-0.875 to -0.125 D	74 (38%)	
Plano	16 (8%)	
+0.125 to +0.875 D	42 (21%)	
+1.00 to +1.875 D	20 (10%)	
≥ +2.00 D	8 (4%)	
Astigmatism category		
None	39 (20%)	
<1.00 D	81 (41%)	
1.00 to 2.00 D	62 (31%)	
≥ 2.00 D	15 (8%)	
SE		-0.28 (-0.53 to -0.05)
J_0_		-0.26 (-0.33 to -0.19)
J_45_		-0.04 (-0.07 to -0.02)
Nidek autorefraction		
SE		-0.12 (-0.41 to 0.16)
J_0_		-0.31 (-0.39 to -0.22)
J_45_		-0.08 (-0.12 to -0.04)
3nethra autorefraction		
SE		0.14 (-0.15 to 0.43)
J_0_		-0.39 (-0.50 to -0.29)
J_45_		0.001 (-0.06 to 0.06)

D = diopters; J_0_ = vertical Jackson cross power in diopters; J_45_ = oblique Jackson cross power in diopters; SE = spherical equivalent power in diopters

**Fig 1:**
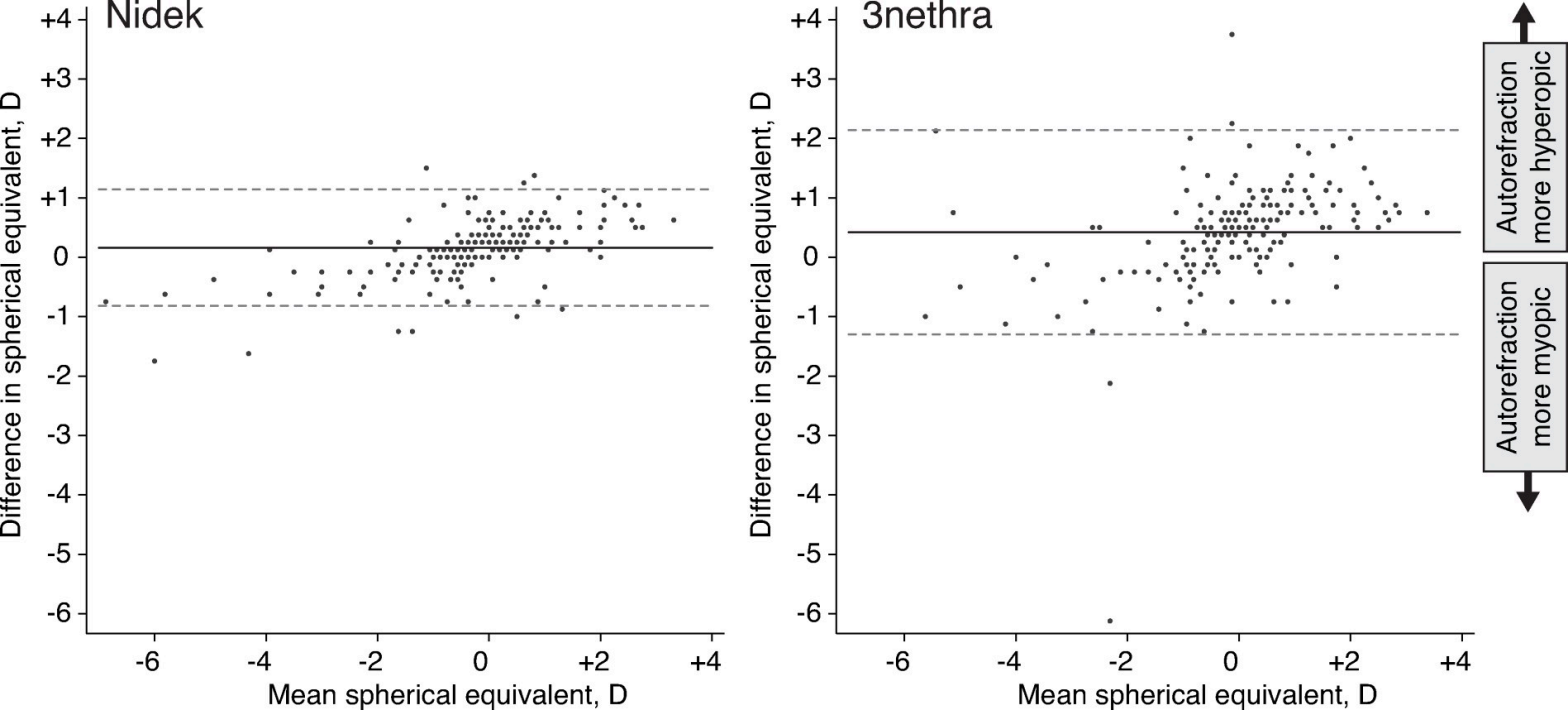
Bland-Altman plots with 95% limits of agreement for (A) subjective refraction and 3Nethra autorefractor and (B) subjective refraction and Nidek autorefractor.

Bland-Altman plots comparing measurements of spherical equivalent between each autorefractor and subjective refraction are shown in Fig 1. Both autorefractors produced spherical equivalent estimates that were on average more hyperopic than subjective refraction (Table 2), with a measurement bias of +0.16 D (95%CI +0.09 to +0.23D) for Nidek and +0.42 D (95%CI +0.28 to +0.54D) for 3nethra (*P*<0.01 for each pairwise comparison). The Nidek autorefractor had narrower 95% limits of agreement for spherical equivalent measurements (-0.82 to +1.14 for Nidek and -1.30 to +2.14 for 3nethra), higher agreement based on estimates of ICC (0.95, 95%CI 0.93–0.96 for Nidek and 0.84, 95%CI 0.80–0.88 for 3nethra), and fewer extreme outliers than the 3nethra (Fig 1). Relationships for astigmatic components were similar, although the magnitude of the differences was smaller (Table 2).

**Table 2:** Mean difference and 95% limits of agreement (LOA) between measurements from subjective refraction and autorefraction.

	Nidek autorefractor minus Subjective	3nethra autorefractor minus Subjective
Component	Mean (95% CI)	Mean (95% CI)
SE		
Mean Difference, D	0.16 (0.09 to 0.23)	0.42 (0.28 to 0.54)
LOA-Upper, D	1.14 (1.00 to 1.30)	2.14 (1.76 to 2.67)
LOA-Lower, D	-0.82 (-1.02 to -0.65)	-1.30 (-1.99 to -0.85)
ICC*	0.95 (0.93 to 0.96)	0.84 (0.80 to 0.88)
J_0_		
Mean Difference, D	-0.05 (-0.09 to -0.01)	-0.13 (-0.21 to -0.06)
LOA-Upper, D	0.48 (0.35 to 0.65)	0.90 (0.66 to 1.20)
LOA-Lower, D	-0.58 (-0.73 to -0.46)	-1.17 (-1.50 to -0.93)
ICC*	0.83 (0.78 to 0.87)	0.55 (0.45 to 0.65)
J_45_		
Mean Difference, D	-0.04 (-0.06 to -0.01)	0.04 (-0.01 to 0.10)
LOA-Upper, D	0.30 (0.27 to 0.35)	0.87 (0.77 to 0.98)
LOA-Lower, D	-0.38 (-0.44 to -0.33)	-0.78 (-0.92 to -0.67)
ICC*	0.81 (0.75 to 0.85)	0.39 (0.28 to 0.51)

D = diopters; J_0_ = vertical Jackson cross power in diopters; J_45_ = oblique Jackson cross power in diopters; SE = spherical equivalent power in diopters.

*Intraclass correlation coefficient (ICC) measuring absolute agreement (one-way random effects model for individual measurements; bootstrapped 95% confidence intervals with resampling at the person level)

**Fig 2:**
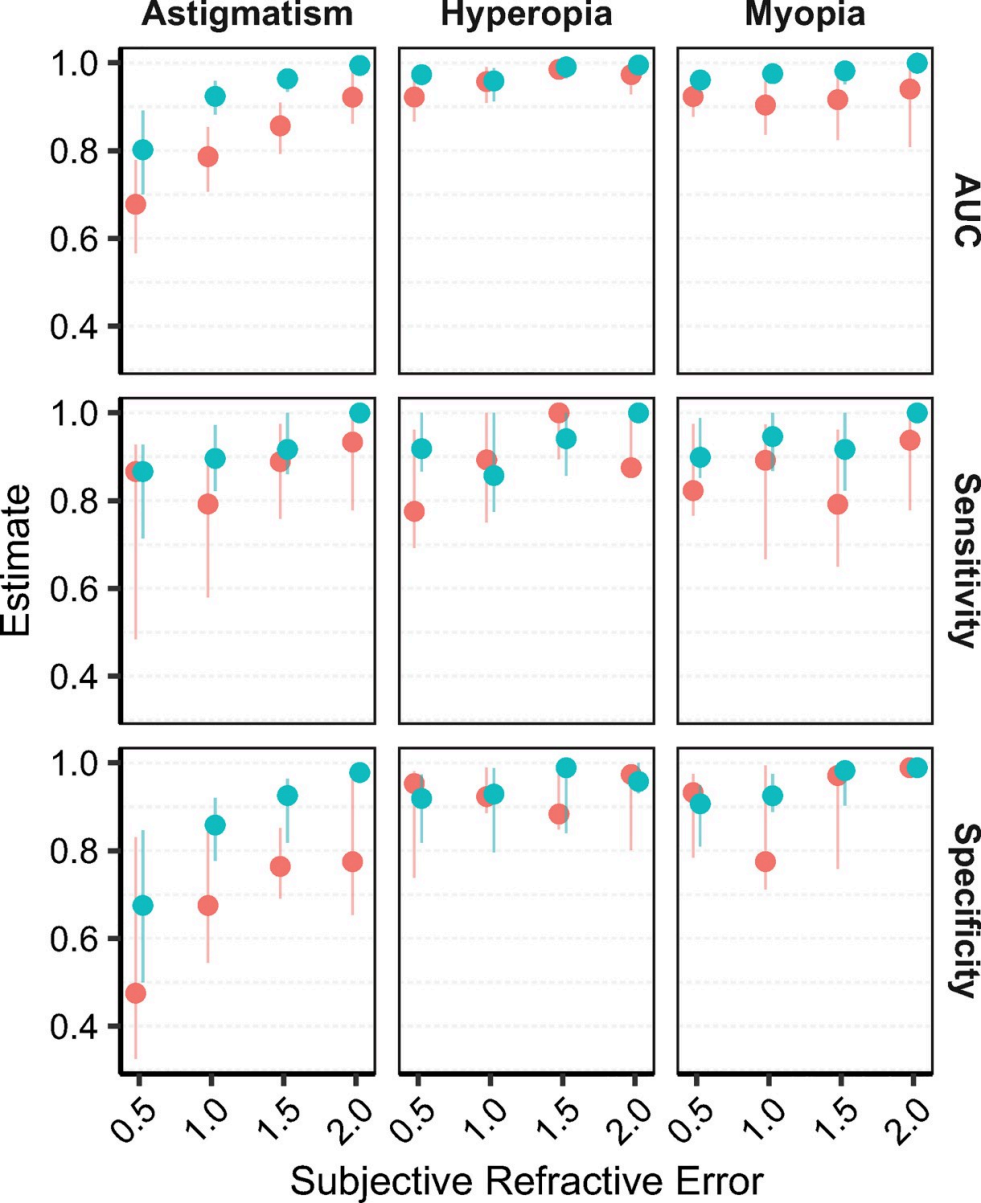
Area under the receiver operating characteristics (ROC) curve, sensitivity, and specificity plotted for each half-diopter of myopia, hyperopia, and astigmatism as assessed from the subjective refraction reference standard. Sensitivities and specificities were assessed from the optimal test threshold for each ROC curve, as assessed by the Youden index. The dots represent the estimate and the lines the 95% confidence interval (green = Nidek, red = 3nethra).

We created ROC curves to calculate the amount of diagnostic information each autorefractor provided over a range of possible levels of refractive error, using subjective refraction as the reference standard. We used the Youden index to determine the autorefractor threshold that optimized sensitivity and specificity for each ROC curve (Fig 2). Diagnostic information, as assessed from the area under the ROC curve, was generally greater for the Nidek than the 3nethra autorefractor under several different reference standard thresholds (Fig 2, top row). Although the optimal sensitivity and specificity of each autorefractor generally increased as the definition of refractive error was made more extreme, the Nidek machine had higher sensitivity (Fig 2 middle row) and specificity (Fig 2 bottom row) across most of the tested thresholds of refractive error. For example, the sensitivity of the Nidek was 94.6% for detection of 1 diopter of myopic spherical equivalent, 85.7% for 1 diopter of hyperopic spherical equivalent, and 89.6% for 1 diopter of astigmatism; corresponding values for the 3Nethra were 89.2%, 89.3%, and 79.2% (Table 3). The specificity of the Nidek was 92.5% for detection of 1 diopter of myopic spherical equivalent, 92.9% for hyperopic spherical equivalent, and 85.8% for astigmatism, with corresponding values of 77.5%, 92.3%, and 67.5% for the 3nethra. The largest differences between the two devices in terms of diagnostic accuracy appeared to be the sensitivity of myopia diagnosis and specificity of astigmatism diagnosis, with the Nidek having higher diagnostic accuracy in each case.

**Table 3:** Sensitivity and specificity of two autorefractors relative to subjective refraction. The optimal test threshold for each index test relative to each reference standard was assessed from the Youden index of the respective receiver operating characteristics (ROC) curve. Diagnostic accuracy was assessed in 197 eyes from 104 people aged 50 years or older.

Reference standard	Optimal threshold of index test	Sensitivity (95%CI)	Specificity (95%CI)
Myopia ≥1D			
Nidek	≥ 0.875 D	94.6% (86.8–100%)	92.5% (88.9–97.5%)
3nethra	≥ 0.25 D	89.2% (66.7–97.4%)	77.5% (71.2–99.4%)
Hyperopia ≥1D			
Nidek	≥ 1.125 D	85.7% (77.4–100%)	92.9% (79.6–98.8%)
3nethra	≥ 1.5 D	89.3% (75.0–100%)	92.3% (88.6–98.9%)
Astigmatism ≥1D			
Nidek	≥ 1.25 D	89.6% (82.1–97.2%)	85.8% (77.7–92.0%)
3nethra	≥ 1.5 D	79.2% (58.0–90.3%)	67.5% (54.5–86.8%)

Myopia and hyperopia indicate measurements of spherical equivalence, in diopters (D)

## Discussion

Compared to subjective refraction, both autorefractors resulted in slightly more hyperopic measurements of spherical equivalent in this study, with 3nethra measurements about one-quarter more hyperopic than Nidek measurements. When comparing pairs of measurements from autorefraction and subjective refraction, the limits of agreement were approximately ±1D for the Nidek machine and ±1.75D for the 3nethra. The sensitivity and specificity of autorefraction for diagnosing refractive error was higher for greater magnitudes of refractive errors. The optimal autorefractor thresholds that optimized the sensitivity and specificity of refractive error detection were different for the two autorefractors, suggesting they could not be used interchangeably for screening purposes.

The limits of agreement measured in this study indicate that 95% of the time, a measurement from the Nidek autorefractor will be within approximately 1D of the measurement obtained from subjective refraction. Prior published studies have reported limits of agreement between the Nidek machine and subjective refraction ranging from ±0.28 D to ± 0.78 D [3–5, 21]. However, these previous studies have either been done on younger populations or did not state the age of participants. It is possible that autorefraction is less accurate in older patients due to media opacities such as cataract or changes in the index of refraction of the lens with age (e.g., index myopia), or due to differences in the ways older adults experience subjective refraction. A study using a more recent version of the Nidek device, the ARK-1, on an adult population found a 95% LOA of -0.80 to +0.69, which, while broader than previous studies of pediatric populations, was still less than what was observed in the present study [22]. It is possible that future versions of the device may demonstrate even better agreement with subjective refraction.

The role of autorefraction in adult vision screening programs is not clear. Adults are more at risk for cataract and posterior segment disease, and while the refraction can give clues about posterior segment disease (e.g., the transient hyperopic shift in central serous chorioretinopathy), the most common causes of visual impairment would not be captured with autorefraction. Complete eye examinations with slit lamp biomicroscopy and indirect ophthalmoscopy would be ideal in order to be able to capture all pathologies, but in resource-limited settings it may make more sense to task-shift eye disease screening to less-skilled, non-ophthalmologic personnel, and reserve the ophthalmologist for the management of complex eye disease and surgery. Moreover, refractive error is still a leading cause of low vision and blindness even among adults, and thus a comprehensive program for eye disease screening would do well to include at least some testing for refractive error [23]. Less expensive instruments like the pinhole occluder have been shown to be sensitive and specific for detection of refractive error, so it may not be necessary to include an expensive electronic device in screening programs [24, 25]. On the other hand, older adults sometimes have difficulty navigating the pinhole test, whereas the autorefractor does not require much cooperation to provide a result. And if an ultimate goal is to provide ready-made spectacles at the point of screening without a trained refractionist, then only an autorefractor would suffice. Because older adults are less able to accommodate during a refraction than children, they need not undergo cycloplegia and they are less likely to have hyperopia underestimated. Thus autorefraction could be a quick and simple screening procedure for adults. The results of the present study suggest that autorefractors may reach a level of diagnostic accuracy that could be useful in a program that wished to dispense ready-made spectacles, even if used as a starting point for the patient to subjectively compare several pairs of eyeglasses [26–29].

Although we used subjective refraction as the gold standard in the present study, subjective refraction is not a perfect comparator [30]. For example, a study that compared subjective refraction performed by two different clinicians found a 95% limits of agreement of ± 0.78 D, which was two times the limits of agreement the same study found for autorefractor repeatability [31]. Moreover, subjective refraction, while based on technician-performed retinoscopy, differed from the autorefractors in that it required cooperation and participation of the test-taker. Nonetheless, we purposefully chose the subjective refraction and not the retinoscopy results as the reference standard because we were most interested in determining whether autorefraction could provide a result similar to the ultimate eyeglasses prescription that would be dispensed.

Strengths of the study include a relatively large sample of eyes, an older population not frequently studied in similar studies, and a realistic clinical setting. Several limitations of this study should also be noted. The autorefractors were relatively old models but we did not have access to newer devices. We did not assess the repeatability of measurements from the same autorefractor, and likewise did not assess inter-rater reproducibility since a single optometrist performed all testing. The study could not easily be masked. We did not randomize the sequence of testing and performed subjective subjective refraction as the final test, which could have produced biased measurements if participants were fatigued at the time of the subjective refraction—though we think this unlikely given the short duration of the autorefraction measurements. We did not perform clinical observations of cataract so could not perform analyses stratified on this factor. The generalizability of the results for populations with different levels of ocular pigmentation, more severe refractive error, or differences in cataract severity is uncertain, as is the generalizability to other models of autorefractor [32].

In conclusion, the sensitivity and specificity of the Nidek autorefractor seemed sufficient for refractive error screening for community outreach programs. However, the relatively wide limits of agreement suggest that the best use of autorefraction may be either as a quick method to identify patients who would benefit from spectacles, or as a starting point for a patient to compare several pairs of read-made spectacles.

## Supporting information

S1 Dataset (CSV)
